# Changes to transform

**DOI:** 10.1590/2177-6709.23.5.014-015.edt

**Published:** 2018

**Authors:** Flávia Artese

**Affiliations:** 1 Universidade do Estado do Rio de Janeiro, Departamento de Odontologia Preventiva e Comunitária (Rio de Janeiro/RJ, Brazil).

In the last years, the bibliometric indexes for the DPJO have grown exponentially,^1^ fruit of the work of the editorial board, as well as of the organization in the publication schedule. To speed up this process, one of the most recent changes was the creation of an *On-line Article* session: in the printed version of the DPJO (in Portuguese only) we include the abstract of two articles, while the full text is found in the digital version. In the current issue, one of these articles is entitled *“Periodontal health knowledge and awareness among subjects with fixed orthodontic appliance”,* written by Jordanian researchers. They evaluated 297 patients using self-administered questionnaires, and concluded that only 8% of them knew what a bacterial plaque is. Additionally, they observed that the treatment duration negatively affected patients’ attitude towards their awareness of periodontal health. 

In 2013, the DPJO became the official journal of the Brazilian Board of Orthodontics and since then it has included in its issues a case report presented by a diplomate for this certification. At the beginning of this year, these case reports were enriched with a broader literature review and a deeper discussion on the main aspects of the case. In the present issue, the need for tooth extractions in orthodontics is discussed, when there is clear indication, and a case is presented with second premolar extractions for crowding solving, but with no incisor retraction, thereby maintaining the facial profile. Not only aesthetic improvement was achieved, but also adequate function, which can be appreciated 5 years post-treatment.

Drawing on these two pieces of information, namely, the lack of motivation of patients with oral hygiene in long treatments and the dread of tooth extraction, I would like to make an association with the reality of Brazilian orthodontics. Patient retreatment after long orthodontic treatments due to absolutely inadequate diagnosis is indeed quite common. But to deal with such a worn out patient and offer him the possibility of yet another treatment, sometimes with extractions, is not an easy task. It demands a high level of confidence on the part of the professional and there is no room for mistakes.

I do not know the number of orthodontic retreatments in the country, but we are aware of the disparate levels of orthodontic education and training of our professionals. Also, we are experiencing a phenomenon very similar to what occurs in other countries, with the growth of mass care in corporate clinics and the ensuing reduction in the individualized character of patient care. This scenario that demands trust is not exclusively ours and was well described by Jorgensen in an editorial for the AJO-DO^2^ regarding the changes implemented by the American Board of Orthodontics. Brazilian orthodontics is proud to have the Brazilian Board of Orthodontics, an institution with the main purpose of promoting professional improvement and to value the excellency in professional practice. The certification of an individual professional may represent much more than a change in his own career. In fact, it may have an important impact on society, by improving clinical care and raising the level of trust in our specialty.^3^

After all, according to Plato, *“let him that would move the world first move himself”*.

Good readings!

Flavia Artese - editor-in-chief (flaviaartese@gmail.com)
